# Targeting LTBP2 Reveals a Novel Anti-Cardiac Remodeling Mechanism of Finerenone Against Doxorubicin-Induced Cardiotoxicity

**DOI:** 10.3390/biom15121703

**Published:** 2025-12-05

**Authors:** Heng Zhang, Nan Zhao, Saiyang Xie, Lanlan Li, Xiaofeng Zeng, Shasha Wang, Ling Yan, Bo Shen, Wei Deng

**Affiliations:** 1 Department of Cardiology, Renmin Hospital of Wuhan University, Wuhan 430060, China; hengzhang98@whu.edu.cn (H.Z.); 2018305231027@whu.edu.cn (N.Z.); yangsai1995@whu.edu.cn (S.X.); 2022203020040@whu.edu.cn (L.L.); lingyan@whu.edu.cn (L.Y.); 2 Hubei Key Laboratory of Metabolic and Chronic Diseases, Wuhan 430060, China; 3 Cardiovascular Research Institute, Wuhan University, Wuhan 430060, China; 729330109@qq.com (X.Z.); 326361992@qq.com (S.W.)

**Keywords:** Finerenone, doxorubicin, cardiotoxicity, cardiac remodeling, LTBP2

## Abstract

Despite the clinical efficacy of doxorubicin (DOX), effective strategies to prevent its cardiotoxicity are still lacking. Finerenone, a nonsteroidal mineralocorticoid receptor antagonist (MRA), has demonstrated cardioprotective properties; however, its role and mechanism in DOX-induced cardiotoxicity (DIC) remain unclear. In this study, Finerenone treatment was found to significantly alleviate DOX-induced cardiac dysfunction and pathological remodeling in both mouse models and cultured cells. Mechanistically, molecular docking suggests that Finerenone may directly bind to Latent Transforming Growth Factor Beta Binding Protein 2 (LTBP2), a key regulator of TGF-β bioavailability. This potential binding could inhibit the LTBP2–TGF-β axis, thereby suppressing DOX-induced activation and subsequent Smad3 phosphorylation. The importance of this pathway was supported by the similar anti-fibrotic effects observed with the TGF-β inhibitor LY2109761. However, our findings on the direct binding of Finerenone to LTBP2 are preliminary and require further validation through additional experimental approaches. These results identify LTBP2 as a novel direct target of Finerenone and reveal an additional mechanism underlying its cardioprotective action, suggesting its potential repurposing for the prevention of DIC.

## 1. Introduction

Doxorubicin (DOX) is a highly effective broad-spectrum chemotherapeutic agent, yet its clinical utility is severely limited by dose-dependent cardiotoxicity [1]. A central pathological feature of DOX-induced cardiotoxicity (DIC) is maladaptive cardiac remodeling, driven primarily by pathological myocardial fibrosis. This process, characterized by cardiomyocyte damage, excessive extracellular matrix deposition, and a decline in systolic function, can lead to arrhythmias and progressive heart failure [2,3,4]. Importantly, DIC-induced cardiac remodeling is largely irreversible [5,6,7]. Current treatments targeting inflammation, oxidative stress, and pro-fibrotic pathways show limited effectiveness against DIC-specific remodeling and often cause significant side effects [8,9,10,11]. Therefore, identifying new and reliable treatment strategies for DIC remains critically important.

Finerenone, a novel nonsteroidal mineralocorticoid receptor antagonist (MRA), has demonstrated potent anti-fibrotic and cardioprotective benefits in clinical and preclinical studies [12,13,14,15]. By blocking the mineralocorticoid receptor, it mitigates inflammatory and fibrotic processes, thereby reducing the risk of cardiovascular events [16,17]. Additionally, Finerenone has been found to lower the incidence of myocardial infarction and vascular inflammation, consequently reducing the risk of heart failure, cardiovascular events, and related mortality [18].

Although growing evidence supports Finerenone’s therapeutic potential, its specific role and molecular mechanisms in alleviating DIC-induced cardiac remodeling are not well defined. While its benefits are mainly linked to mineralocorticoid receptor (MR) blockade, it remains uncertain whether other mechanisms beyond MR antagonism contribute to its protection against DIC. This study therefore aims to clarify not only the protective effects of Finerenone on DIC-induced cardiac remodeling but also the involved molecular pathways, which may uncover a new pharmacological dimension of Finerenone for clinical prevention of DIC.

## 2. Materials and Methods

### 2.1. Experimental Animals

All animal procedures were conducted in accordance with the NIH Guide for the Care and Use of Laboratory Animals and approved by the Animal Ethics Committee of Renmin Hospital of Wuhan University (Approval No: WDRM-20230408A). Eight-week-old male C57BL/6J mice were obtained from the Chinese Academy of Medical Sciences (Beijing). Finerenone (TargetMol, CAS 1050477-31-0) was dissolved in DMSO and diluted with saline to ≤5% DMSO (*v*/*v*) for oral gavage at 5 mg/kg [19], while doxorubicin (TargetMol, CAS 23214-92-8) was dissolved in sterile water (2 mg/mL) and administered intraperitoneally at 5 mg/kg [20]. After one week of acclimation, mice were randomly assigned to four groups: Saline control, Finerenone alone, DOX alone, and DOX + Finerenone combination (n = 8 per group). The sample size was determined a priori using G*Power software (version 3.1.9.7) based on an anticipated large effect size (d = 1.8), α = 0.05, and statistical power of 0.95. To account for potential attrition, we initially allocated 10 mice per group, with two excluded from each group based on pre-defined criteria, resulting in the final n = 8. Finerenone was administered weekly by gavage, with the DOX and DOX + Finerenone groups receiving weekly DOX injections for four weeks. In the combination group, Finerenone was administered 30 min prior to DOX. All assessments (body weight, echocardiography, and blood collection) were performed in week 5 by investigators blinded to group assignments, followed by cervical dislocation.

### 2.2. Echocardiography

As per our previous study [21], transthoracic echocardiography was performed in week 5 on mice anesthetized with 1.5% isoflurane. Cardiac function was assessed using a high-resolution imaging system (VisualSonics Vevo 3100, FUJIFILM VisualSonics Inc., Toronto, ON, Canada) equipped with an MX550D linear array transducer (40 MHz). The obtained parameters included left ventricular ejection fraction (LVEF), fractional shortening (LVFS), end-systolic and end-diastolic volumes (LVESV, LVEDV), systolic posterior wall thickness (LVPWs), heart rate (HR), early-to-late diastolic inflow ratio (E/A), and early diastolic transmitral velocity-to-tissue velocity ratio (E/E).

### 2.3. Histological Analysis

As previously described [22,23], hearts were fixed in 4% paraformaldehyde, paraffin-embedded, and cut into 5 µm sections. H&E, Masson’s trichrome and Picro Sirius Red staining were performed following the manufacturer’s instructions. For immunofluorescence, sections were incubated overnight with primary antibodies, washed with PBS, treated with secondary antibodies, mounted in DAPI-containing medium and viewed under a fluorescence microscope.

### 2.4. Biochemical Analyses

Markers of myocardial injury, including cardiac troponin T (cTnT) activity, cardiac creatine kinase isoenzyme (CK-MB), and lactate dehydrogenase (LDH) levels, as well as fibrosis-related markers such as N-terminal B-type natriuretic peptide precursor (NT-proBNP), transforming growth factor β (TGF-β), and tumor necrosis factor-α (TNF-α), were measured in serum samples using commercially available kits (Bioswamp, MU30030, MU30071, MU30252, BTK147, MU30025, MU30772, Wuhan Bainle Biotechnology Co., Ltd., Wuhan, China), following the manufacturer’s protocol. Blood was collected from the orbital venous plexus of mice at the endpoint, allowed to clot at room temperature, and then centrifuged at 3000× *g* for 15 min to obtain serum.

### 2.5. Serum Potassium Determination

**Figure 1:**
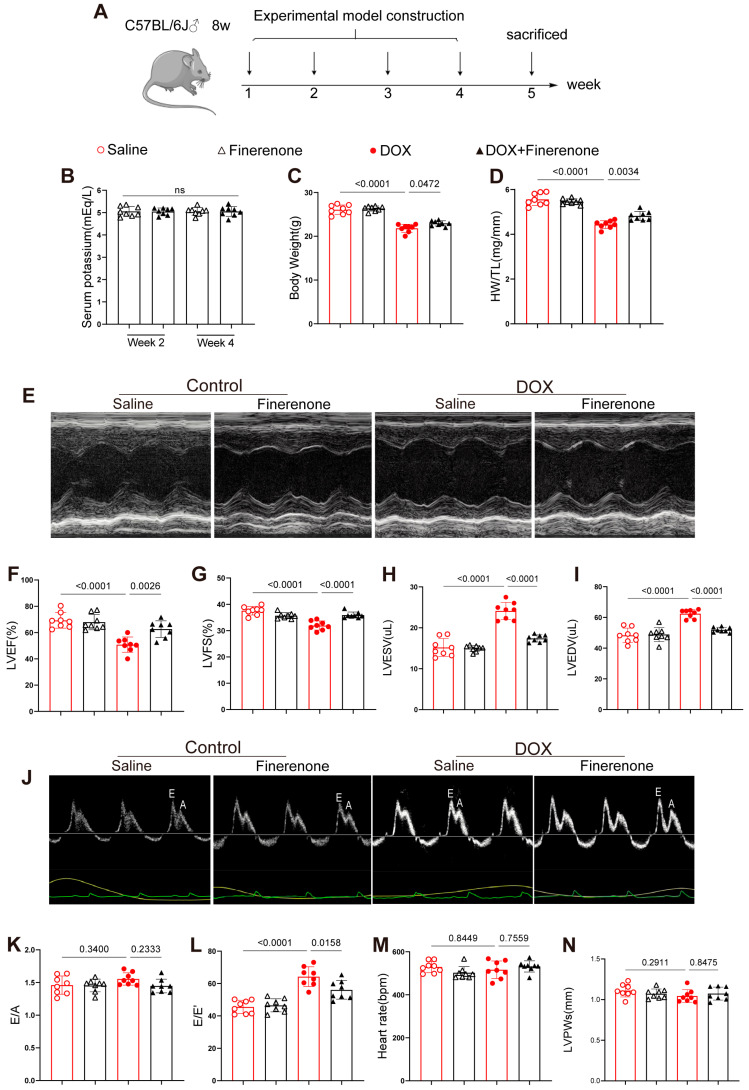
Effect of Finerenone on body weight, HW/TL and cardiac function in DIC mice. (**A**) Experimental protocol. (**B**) Serum potassium concentrations in Finerenone-treated and DOX + Finerenone-treated groups at weeks 2 and 4 post-intervention. (**C**) Comparative analysis of body weight among experimental groups upon model completion. (**D**) Statistical evaluation of heart weight-to-tibia length (HW/TL) ratios across experimental groups. (**E**) Representative M-mode echocardiographic tracings illustrating cardiac structural dynamics. (**F**) Quantitative echocardiographic analysis of left ventricular ejection fraction (LVEF). (**G**) Echocardiographic assessment of left ventricular fractional shortening (LVFS). (**H**) Left ventricular end-systolic volume (LVESV) measurements derived from echocardiography. (**I**) Left ventricular end-diastolic volume (LVEDV) quantification via echocardiographic analysis. (**J**) Representative pulse-wave Doppler echocardiographic images for diastolic function evaluation (E wave, A wave). (**K**) Echocardiographic comparison of E/A ratios among groups. (**L**) Analysis of E/E’ ratios as an index of diastolic dysfunction. (**M**) Heart rate (HR) measurements. (**N**) Echocardiographic quantification of left ventricular posterior wall thickness at systole (LVPWs). All results are shown as the mean ± SEM, with each data point representing a mouse (n = 8); *p*-values are indicated.

Tail vein blood was collected from mice in the 2nd and 4th weeks, and serum was obtained by centrifugation. For the serum potassium measurement, 50 μL of serum was added to an EP tube, followed by 450 μL of reagent one working solution. The mixture was thoroughly vortexed and incubated at room temperature (25 °C). After incubation, it was centrifuged at 8000 rpm for 10 min. The resulting supernatant was collected for analysis. The absorbance of the supernatant was measured at 520 nm using a spectrophotometer that had been preheated for at least 30 min. The instrument was calibrated with distilled water prior to use. All procedures were performed according to the manufacturer’s instructions (BC2775, Beijing Solarbio Science & Technology Co., Ltd., Beijing, China). The data are presented in Figure 1B.

### 2.6. Cell Culture and Processing

Neonatal rat cardiac fibroblasts (NRCFs) were isolated from 1–3-day-old rats as previously described [24,25,26] and used before passage 2 to avoid spontaneous transdifferentiation. HUVECs (YRGene, NC006, Changsha Yinrun Biotechnology Co., Ltd., Changsha, Hunan, China) and NRCFs were cultured in DMEM with 10% FBS and 1% penicillin–streptomycin at 37 °C and 5% CO_2_. Prior to treatments, cells at 70–80% confluence were serum-starved for 12 h. Finerenone and DOX were prepared as 10 mM stocks in DMSO and water, respectively, and diluted to working concentrations in culture medium (Finerenone: 1–20 μM; DOX: 1 μM [27]). The final DMSO concentration was kept at ≤0.1% to minimize solvent effects. For cytotoxicity assessment, HUVECs seeded in 96-well plates (4 × 10^4^ cells/well) were treated with Finerenone (0–10 μM) with or without 1 μM DOX for 24 h to evaluate acute effects. Viability was measured using the CCK-8 assay. Based on dose–response results, 5 μM Finerenone was selected for subsequent experiments due to its optimal balance between efficacy and minimal cytotoxicity. NRCFs were treated with 1 μM DOX ± 5 μM Finerenone for 24 h to investigate the effects on fibroblast behavior. In experiments where the TGF-β pathway was targeted, 10 μM LY2109761 [28] was added for the final 2 h to assess the specific role of this pathway inhibitor.

### 2.7. Wound-Healing Assay

The same method as a previous study was used [25]. NRCFs were incubated in 6-well plates until reaching 90% confluence, after which a wound was created by dragging 10 μL pipette tips along the center of the plate. The cells were then washed twice with PBS, and serum-free medium was added. After 24 h of incubation, images were captured, and cell migration was assessed. The migration rate was quantified by comparing the cell coverage to the initial cell-free gap.

### 2.8. Real-Time Quantitative PCR

After extraction of total RNA using Trizol (Invitrogen Corporation, 15596026, Carlsbad, CA, USA), the RNA was reverse transcribed to complementary DNA (cDNA) using the cDNA synthesis kit (Roche, 489703001, Basel, Switzerland). The indicated genes were amplified using LightCycler 480 SYBR Green 1 Master Mix (Roche, 04887352001, Basel, Switzerland), and the relative mRNA level is normalized to β-actin (Col1, Col3, Snail1, Snail2, Twist1, Twist2). Please refer to Table 1.

### 2.9. RNA-Seq Library Construction and Sequencing

RNA-seq was performed by Wuhan IGENEBOOK Biotechnology Co (Wuhan, Hubei, China). Briefly, total RNA was extracted using the Trizol method. All the RNA samples were assessed for their integrity using the Qsep400 instrument. To construct RNA libraries with the VAHTS mRNA-seq V8 Library Prep Kit (Novogene, Beijing, China)for Illumina, 1 μg of total RNA was used [29]. The procedure included polyA-selected RNA extraction, RNA fragmentation, random hexamer-primed reverse transcription, and 150nt paired-end sequencing by Illumina Novaseq 6000(Illumina, Inc., San Diego, CA, USA).

### 2.10. Western Blot

Total protein was extracted from mouse hearts and cultured NRCFs using RIPA lysis buffer (Servicebio, g2002, Wuhan, Hubei, China) containing a mixture of protease inhibitors. Protein expression was detected using a BCA protein assay kit (Thermo Fisher Scientific, 23227, Waltham, MA, USA) to determine protein concentration. Proteins were then separated by 10% SDS-PAGE and transferred to a PVDF membrane (Micropore, fl00010, 3M Company, St. Paul, MN, USA), which was blocked with 5% nonfat milk for 1 h. The washed membranes were further incubated with anti-LTBP2 (Santa Cruz, sc-166199, Dallas, TX, USA), anti-TGF-β (HUABIO, HA721143, Hangzhou, Zhejiang, China), Smad3 (HUABIO, ET1607-41, China), p-Smad3 (HUABIO, ET1609-41, China), Col I (ABclonal, A22090, Wuhan, Hubei, China), Col III (HUABIO, HA720050, China), Vimentin (HUABIO, ET1610-39, China), α-SMA (HUABIO, ET1607-53, China), β-Tublin (ABclonal, A12289, China), β-actin (ABclonal, AC026, China) and GAPDH (ABclonal, A19056, China) with primary antibodies overnight at 4 °C and with secondary antibodies at room temperature for 1 h. The immunoblots were then detected using a chemiluminescence ECL kit (Bio-Rad, Hercules, CA, USA) and visualized by Image Lab5.2.1 software.

### 2.11. Bioinformatics Analysis of RNA-Seq

Raw sequencing reads were first assessed for quality using FastQC (Version 0.12.0). Adapter sequences and low-quality bases were then trimmed using Cutadapt (version 5.0) [30]. The resulting high-quality clean reads were aligned to the mouse reference genome (GRCm39/MM10) using HISAT2 (version 2.2.1) with default parameters. FeatureCounts (version 2.0.1) was used to assign reads to genomic features and generate the raw gene count matrix. Differential expression analysis was performed on the raw count data using the edgeR package (Version 4) In R [31]. Specifically, genes with low expression were filtered out (retaining genes with counts per million (CPM) > 1 in at least n samples, where n is the size of the smallest group). Data was then normalized using the TMM (trimmed mean of M-values) method. A generalized linear model was applied to test for differential expression between the DOX and control groups. Genes with a false discovery rate (FDR) adjusted *p*-value < 0.05 and an absolute log2 fold change > 1 were considered statistically significantly differentially expressed genes (DEGs) [32,33,34,35] enrichment analysis. Terms with a q-value < 0.05 were considered significantly enriched. The complete list of all DEGs has been provided as Supplementary Dataset S1.

### 2.12. Molecular Docking and Result Analysis

Using LeDock software (version 1.0), molecular docking simulations were performed between small molecules and proteins. Through simulation, the binding affinity between the protein and the compound can be evaluated. LeDock Win32 outputs multiple PDB format files, recording the specific posture of different compounds binding to proteins. From there, we screened out the complexes with the highest binding scores and further visualized them using Maestro 13.5 software. In Maestro 13.5, we made detailed use of the “interactions Toggle” tool to label and evaluate key interactions between ligands and receptors and the “2D Sketcher” module to generate a two-dimensional map of ligand and receptor interactions to better understand the interactions between molecules.

### 2.13. Statistical Analysis

GraphPad Prism 9.4.1 software was used for all statistical analyses. The normality of the data distribution was first assessed using the Shapiro–Wilk test. For data satisfying normality and homogeneity of variance, one-way ANOVA was performed, followed by Tukey’s post hoc test for multiple comparisons. For data that were not normally distributed, the non-parametric Kruskal–Wallis test was applied, followed by Dunn’s post hoc test. All data are presented as mean ± SEM. A *p*-value < 0.05 was considered statistically significant.

## 3. Results

### 3.1. Effect of Finerenone on Body Weight, HW/TL and Cardiac Function in DIC Mice

C57BL/6J mice received weekly DOX for 4 weeks to induce chronic cardiotoxicity (Figure 1A). DOX reduced body weight and heart weight/tibia length (HW/TL); both losses were reversed by co-administered Finerenone (Figure 1C,D). Echocardiography showed lower LVEF and LVFS and larger LVESV and LVEDV in DOX mice; Finerenone restored all four parameters (Figure 1E–I). DOX also raised E/E′, a sign of diastolic dysfunction, and Finerenone normalized it (Figure 1L). E/A, HR and LVPWs did not differ among groups (Figure 1J,K,M,N). Thus, Finerenone protects against DOX-induced cardiac dysfunction and was used to study the underlying anti-remodeling and anti-fibrotic mechanisms.

### 3.2. Finerenone Reduces Serum Markers of Myocardial Injury and Fibrosis in DIC Mice

**Figure 2:**
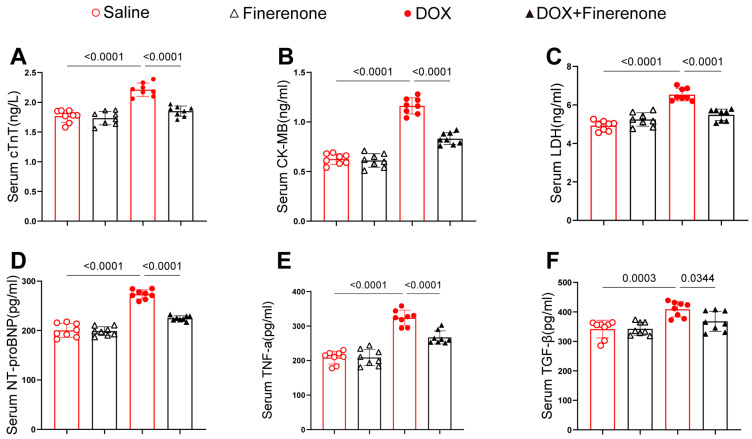
Finerenone reduces serum markers of myocardial injury and fibrosis in DIC mice. (**A**–**C**) Markers of myocardial injury: cTnT, CK-MB, and LDH. (**D**–**F**) Fibrotic markers: NT-proBNP, TNF-α, and TGF-β. Each group was determined by ELISA kits, n = 8. All results are shown as the mean ± SEM; *p*-values are indicated.

To evaluate the impact of Finerenone on myocardial injury and fibrosis in DOX-treated mice, serum levels of cTnT, CK-MB, LDH, NT-proBNP, TNF-α, and TGF-β were measured using ELISA (Figure 2). DOX-treated mice exhibited significantly elevated levels of these markers compared with controls. In contrast, Finerenone treatment markedly reduced their serum concentrations. These findings indicate that Finerenone attenuates the release of key biomarkers associated with myocardial injury and fibrosis in mice with DIC, with the downregulation of TGF-β being of particular interest given its central role in fibrotic pathways.

### 3.3. Finerenone Mitigates Cardiac Injury and Alleviates Cardiac Remodeling in DIC Mice

**Figure 3:**
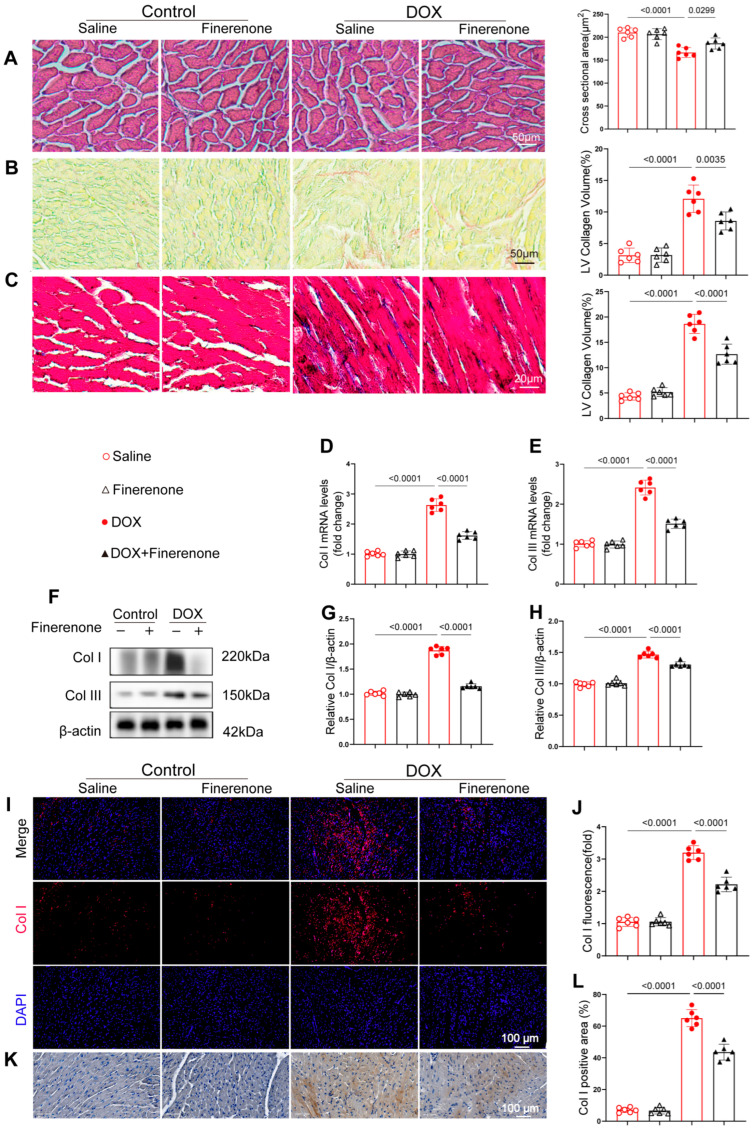
Finerenone mitigates cardiac injury and alleviates cardiac remodeling in DIC mice. (**A**) Representative images of H&E-stained sections and quantification of the cardiomyocytes’ cross-sectional area. (**B**) Picric Sirius red (PSR) staining and quantification of the LV collagen volume. (**C**) Representative cardiac Masson trichrome staining and quantification of the LV collagen volume. (**D**,**E**) PCR was used for mRNA quantitative analysis of Col I and Col III. Normalized to β-actin, n = 6. (**F**–**H**) Western blot analysis and quantification of Col I and Col III, n = 6. (**I**–**J**) Representative images of immunofluorescence staining and quantification of fluorescence intensity for Col. (**K**–**L**). Representative images of immunohistochemistry staining and quantification of positive area for Col I. All results are shown as the mean ± SEM; *p*-values are indicated.

To evaluate the protective effect of Finerenone against DOX-induced myocardial fibrosis, we conducted histopathological and molecular analyses. HE staining revealed well-organized cardiomyocytes in control mice, while DOX treatment caused obvious structural disruption. Finerenone treatment notably improved myocardial architecture (Figure 3A). Both PSR and Masson staining showed substantial collagen deposition in DOX-treated hearts, which was significantly reduced by Finerenone (Figure 3B,C). Consistent with these findings, DOX treatment increased mRNA and protein levels of collagen I and III, and these effects were reversed by Finerenone (Figure 3D–H). Immunofluorescence and immunohistochemical staining further confirmed the elevated collagen I expression after DOX exposure and its reduction following Finerenone intervention (Figure 3I–L). Together, these results demonstrate that Finerenone alleviates DOX-induced cardiac fibrosis and maladaptive remodeling. This strong anti-fibrotic effect suggests that Finerenone may target an upstream regulator of collagen production, such as the TGF-β pathway.

### 3.4. Effect of Finerenone on EndMT and Cardiac Fibroblast Proliferation In Vitro

**Figure 4:**
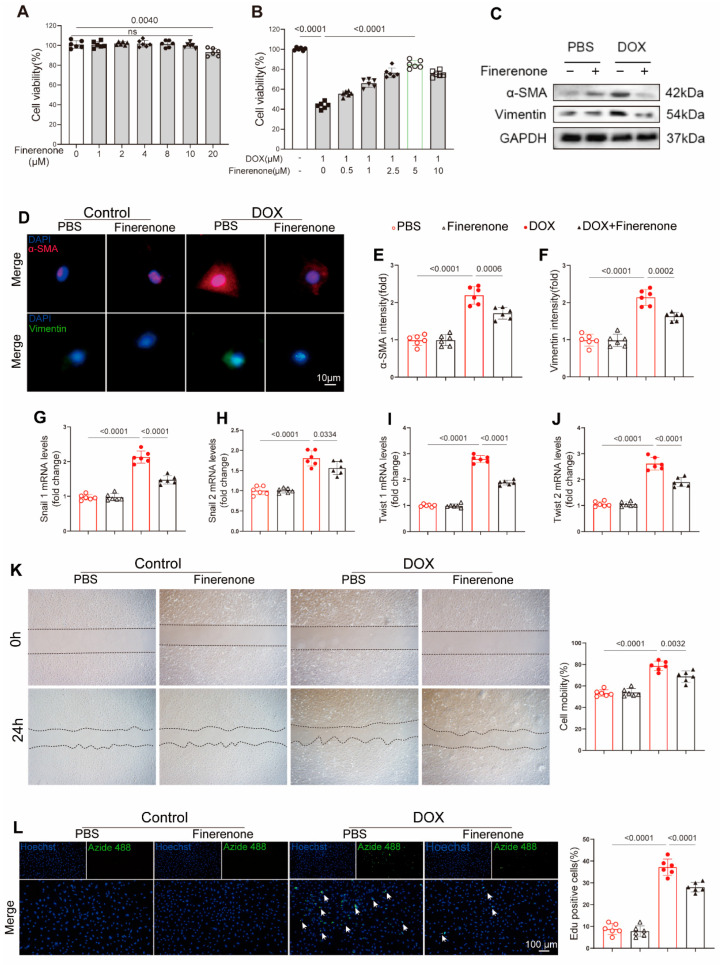
Effect of Finerenone on EndMT and proliferation in vitro. (**A**) Cell viability after treatment with different Finerenone doses, measured by CCK8, n = 6. The horizontal lines indicate a comparison between the two endpoint groups. (**B**) Cell viability after treatment with different Finerenone doses in the presence of DOX, measured by CCK8, n = 6. The horizontal lines indicate a comparison between the two endpoint groups. (**C**) Representative Western blot images showing the expression of α-SMA and vimentin, n = 6. (**D**–**F**) Immunofluorescence staining for α-smooth muscle actin (α-SMA) and vimentin was performed, and the fluorescence intensity of each marker was quantified. (**G**–**J**) PCR was used for mRNA quantitative analysis of twist1, twist2, snial1 and snial2, normalized to β-actin in vitro, n = 6. (**K**) Cell migration capacity was assessed using a scratch assay. (**L**) NRCFs were stained for EdU, and EdU-positive cells were quantified. Hoechst is used to label all nuclei (blue). Azide 488 for Specifically Labeling the Nuclei of Proliferating Cells (Green). All results are shown as the mean ± SEM; *p*-values are indicated.

The CCK-8 assay showed that Finerenone had minimal cytotoxicity in HUVECs at low concentrations. A significant decrease in cell viability was only observed at 20 μM (Figure 4A). Finerenone protected against DOX-induced cell death in a dose-dependent manner, with maximal efficacy at 5 μM. This concentration was selected for subsequent studies (Figure 4B). To investigate the role of Finerenone in endothelial-to-mesenchymal transition (EndMT), an in vitro model of DOX-induced injury was established in HUVECs. Western blot analysis indicated that DOX increased the protein expression of vimentin and α-SMA. These increases were significantly reduced by Finerenone co-treatment (Figure 4C). Similarly, immunofluorescence staining confirmed that DOX exposure elevated vimentin and α-SMA expression, an effect that was markedly suppressed by Finerenone (Figure 4D–F). Furthermore, PCR analysis demonstrated that Finerenone downregulated the mRNA levels of EndMT-associated transcription factors, including Snail1, Snail2, Twist1, and Twist2 (Figure 4G–J). In functional assays using neonatal rat cardiac fibroblasts (NRCFs), Finerenone inhibited DOX-induced cell migration in a scratch-wound assay (Figure 4K) and reduced proliferation, as assessed by EdU staining (Figure 4L). These in vitro findings confirm that Finerenone directly attenuates pro-fibrotic processes. Since EndMT and fibroblast activation are well-known downstream events of TGF-β signaling, we examined whether Finerenone exerts its effects by modulating this pathway.

### 3.5. Finerenone Attenuates Cardiac Remodeling Through Inhibiting the Activation of the LTBP2/TGF-β Pathway

**Figure 5:**
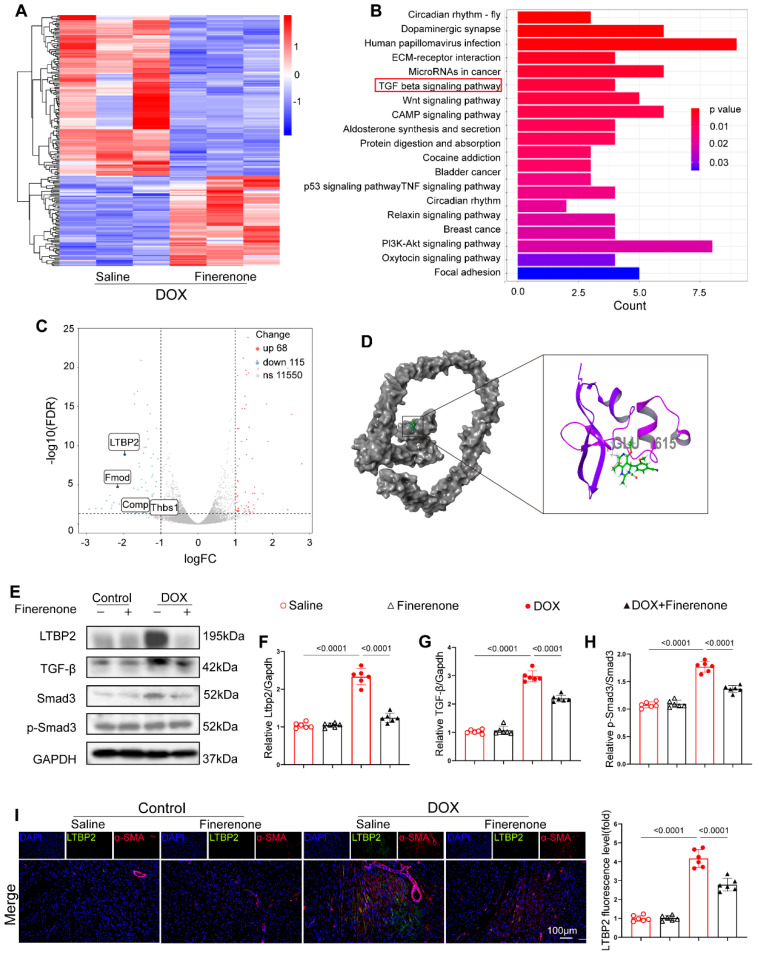
Finerenone attenuates cardiac remodeling through modulating the LTBP2/TGF-β pathway. (**A**) The heat map shows the average normalized expression level. (**B**) KEGG pathway enrichment analysis of the downregulated genes. (**C**) Volcano map shows the enrichment of 4 differentially expressed genes in the TGF-β pathway. (**D**) The cartoon diagram of the binding conformation of Finerenone and LTBP2 protein shows the site of interaction. (**E**) Representative images of Western blot in each group, n = 6. (**F**–**H**) Statistical analysis of relative expression of LTBP2, TGF-β, Smad3, p-Smad3, n = 6. (**I**) Representative images of immunohistofluorescence co-staining of LTBP2 and α-SMA, and the fluorescence level of LTBP2 was quantified. DAPI (blue staining) represented the nucleus, α-SMA (red staining) was used to visualize the cytoskeleton of cardiac fibroblasts, and LTBP2 expression levels in each group were visualized via the corresponding antibody labeled with green fluorescence (green staining). All results are shown as the mean ± SEM; *p*-values are indicated.

To explore how Finerenone alleviates cardiac remodeling in a DIC mouse model, RNA sequencing was performed on cardiac tissues from mice with or without Finerenone treatment. Transcriptomic analysis identified 11,733 differentially expressed genes (DEGs) (Figure 5A) (Supplementary Dataset S1). KEGG pathway enrichment analysis showed significant involvement of the TGF-β pathway (Figure 5B), and GSEA revealed a significant enrichment of the TGF-β signaling pathway (Supplementary Figure S6), which is a key regulator of fibrosis [36]. Key fibrotic genes in this pathway—LTBP2 (latent transforming growth factor beta binding protein 2), Fmod (Fibromodulin), COMP (cartilage oligomeric protein), and Thbs1 (Thrombospondin 1)—were notably enriched. LTBP2 showed the pronounced downregulation (Figure 5C) (Supplementary Figure S5). Molecular docking analysis further suggested a strong binding interaction between Finerenone and the Glu1615 residue of LTBP2 (Figure 5D) (Supplementary Table S1), indicating a direct mechanism of action. Since LTBP2 is known to promote collagen synthesis and fibronectin assembly via TGF-β signaling and ECM regulation [36], we proposed that Finerenone reduces fibrosis through the LTBP2/TGF-β axis. Western blot analysis confirmed that protein levels of LTBP2, TGF-β, and p-Smad3 were increased in DOX-treated mice compared to saline controls. These increases were significantly reduced by Finerenone treatment (Figure 5E–H). Consistent with these results, immunofluorescence co-staining revealed enhanced LTBP2 expression colocalized with α-SMA in DOX-induced mice [37]. This effect was markedly attenuated by Finerenone administration (Figure 5I). Together, these results identify LTBP2 as a novel direct target of Finerenone and demonstrate that the LTBP2/TGF-β/Smad3 axis is a key mechanism by which Finerenone alleviates cardiac remodeling.

### 3.6. Finerenone Regulates the Activation of the DOX-Induced LTBP2/TGF-β Pathway

**Figure 6:**
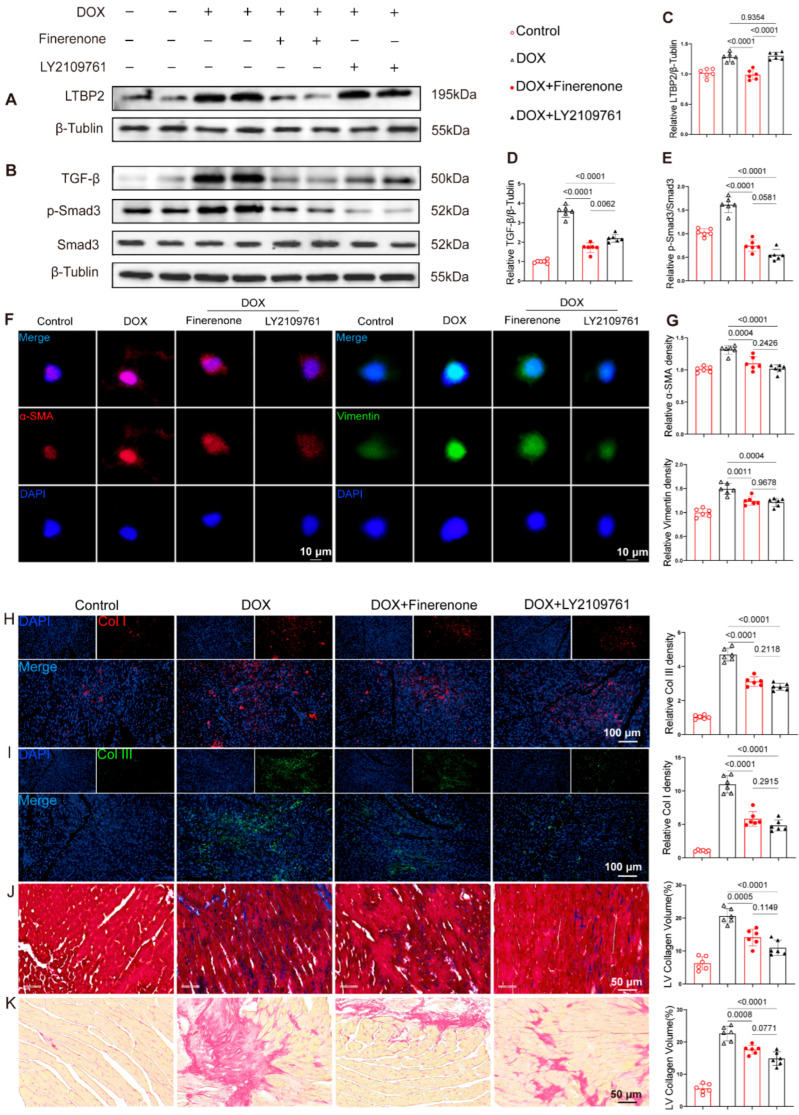
Finerenone regulates the activation of the DOX-induced LTBP2/TGF-β pathway. (**A**,**B**) Representative Western blot images of each group, n = 6. (**C**–**E**) Statistical analysis of relative expression levels of LTBP2, TGF-β, and p-Smad3, n = 6. (**F**,**G**) Representative immunofluorescence staining images and quantitative analysis of α-SMA and Vimentin. (**H**,**I**) Representative tissue immunofluorescence images and quantitative analysis of Col I and Col III. (**J**) Representative Masson staining images and quantitative analysis across groups. (**K**) Representative PSR staining images and quantitative analysis across groups. All results are shown as the mean ± SEM; *p*-values are indicated.

Compared to the PBS group, DOX treatment significantly increased the expression of LTBP2, TGF-β, and p-Smad3. This increase was markedly suppressed by Finerenone co-treatment (Figure 6A–E). Similarly, the TGF-β inhibitor LY2109761 significantly reduced TGF-β and p-Smad3 levels. However, LY2109761 did not significantly affect LTBP2 expression (Figure 6A,C). To test if the anti-fibrotic effect of Finerenone depends on the LTBP2/TGF-β pathway, a rescue experiment was performed using LY2109761. DOX-exposed cells showed increased immunofluorescence signals for α-SMA and vimentin (Figure 6F,G), higher expression of Col I and Col III (Figure 6H,I), and a larger fibrotic area based on Masson and PSR staining (Figure 6J,K). Intervention with LY2109761 reduced these fibrotic markers and collagen deposition to a level similar to Finerenone. No significant differences were observed between the two treatment groups (Figure 6F–K).

## 4. Discussion

**Figure 7:**
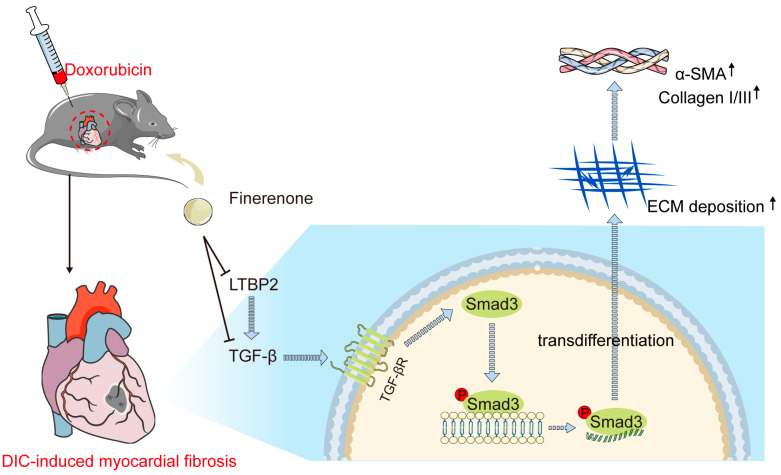
Schematic illustration of this study. DOX significantly increases LTBP2 production and TGF-β activation, which subsequently binds to the TGF-β receptor and stimulates Smad3 phosphorylation, leading to fibrotic phenotypes. Finerenone inhibits LTBP2 secretion and TGF-β/Smad3 pathway activation, thereby reducing DOX-induced cardiac fibroblast proliferation and cardiac remodeling.

This study identifies LTBP2 as a novel target of Finerenone, revealing a previously unrecognized mechanism underlying its cardioprotection against DIC. The findings suggest that Finerenone may directly interact with LTBP2, a key regulator of TGF-β signaling, which plays a central role in cardiac fibrosis and remodeling associated with DIC. This interaction potentially disrupts the LTBP2–TGF-β axis, leading to the inhibition of TGF-β activation and subsequent Smad3 phosphorylation, thereby attenuating cardiac dysfunction and pathological remodeling (Figure 7). The therapeutic potential of Finerenone is further underscored by its comparable effects to the TGF-β inhibitor LY2109761, suggesting that modulation of the LTBP2–TGF-β pathway could be a viable strategy for mitigating DOX-induced cardiac side effects.

In addition to its pro-fibrotic effects, DOX treatment induced a significant reduction in cardiomyocyte cross-sectional area, a hallmark of DIC. This phenomenon is primarily driven by cardiomyocyte atrophy resulting from inhibited protein synthesis and the direct loss of cardiomyocytes via apoptosis, rather than mere displacement by fibrotic tissue [38,39,40,41,42,43]. The observed atrophy reflects a maladaptive remodeling response to the severe energetic stress and cytotoxicity imposed by DOX. Importantly, Finerenone treatment ameliorated this reduction in cell size, underscoring its broader cytoprotective effects that extend beyond the inhibition of fibrotic deposition. This protective action may involve the preservation of anabolic signaling and suppression of cell death pathways, highlighting the multi-faceted cardioprotective profile of Finerenone. The translational significance of our work is significant. These significant findings substantially deepen our understanding of Finerenone’s molecular mechanisms and position LTBP2 as a highly promising candidate for novel anti-fibrotic strategies. The identification of LTBP2 as a direct target offers fresh perspectives for pharmacological intervention against cardiac fibrosis, particularly in cancer therapy. Importantly, as an already approved drug with a known safety profile in cardiorenal diseases, Finerenone holds a significant advantage over novel compounds. This established background could greatly accelerate its repurposing and facilitate smoother integration into cardio-oncology trials and clinical practice. This study thus provides a solid foundation for future research to validate these interactions and explore the clinical application of Finerenone as an adjunctive cardioprotective agent in DOX-based chemotherapy.

However, this study has several limitations that highlight directions for future research. First and most critically, we lack direct biochemical and functional genetic validation of the Finerenone–LTBP2 interaction. Although transcriptomics, molecular docking, and downstream pathway analyses suggest LTBP2 is a direct target, definitive confirmation awaits experiments such as co-immunoprecipitation or mass spectrometry-based pull-down assays. Furthermore, pinpointing the binding site through site-directed mutagenesis (e.g., of the Glu1615 residue) will be a crucial step to verify binding specificity. To definitively establish the causal role of LTBP2, future studies will employ genetic approaches, including LTBP2 knockdown and overexpression, to determine if modulating LTBP2 levels is both necessary and sufficient to replicate Finerenone’s anti-fibrotic effects. Moreover, the precise mechanism by which Finerenone binding leads to reduced LTBP2 levels remains to be fully elucidated. Future studies should employ cycloheximide chase assays to determine if Finerenone directly shortens LTBP2 protein half-life and luciferase reporter assays of the LTBP2 promoter to investigate potential feedback mechanisms on its transcription. Second, the relative contributions of MR-dependent versus independent mechanisms remain unclarified. Although our data support the existence of an MR-independent pathway via LTBP2/TGF-β signaling, definitive clarification requires future verification in MR-knockout models or via head-to-head comparison with classical MRAs (e.g., spironolactone, eplerenone). Third, the potential impact of Finerenone on DOX’s antitumor efficacy was not assessed. Before translating Finerenone into cardio-oncology practice, it is imperative to rigorously evaluate in tumor-bearing animal models that it does not compromise chemotherapy’s anticancer effects. Fourth, it should be noted that our in vivo experiments did not include a dedicated DMSO vehicle control group. Future studies would benefit from including a full vehicle control (saline with ≤5% DMSO) to further confirm the specific effects of Finerenone. Fifth, there are limitations in how well our models reflect clinical reality. Our 4-week mouse model may not fully recapitulate the chronic, cumulative cardiotoxicity seen in patients. Optimizing the combination dosing regimen of Finerenone with chemotherapeutic agents (including timing and sequence) to better mirror clinical practice is an important future direction. Finally, to fully assess its therapeutic potential, future studies should test the long-term cardioprotective effects of Finerenone in chronic models; validate the efficacy and mechanism of Finerenone in female animal models to fully assess the influence of sex on its cardioprotective benefits; and explore the upstream transcriptional regulators of LTBP2 to gain deeper insights into its transcriptional regulatory network.

In conclusion, our work provides a compelling preclinical rationale for repurposing Finerenone as a cardioprotective co-adjuvant in oncology. Future studies should prioritize the direct validation of the Finerenone–LTBP2 interaction through co-immunoprecipitation and the assessment of its impact on anticancer therapy, which are essential steps toward clinical application.

## 5. Conclusions

This study shows that the nonsteroidal mineralocorticoid receptor antagonist Finerenone protects against DOX-induced cardiac remodeling and dysfunction. A novel mechanism for this protection was identified: molecular docking indicates that Finerenone may directly bind to the Glu1615 residue of LTBP2, which could inhibit the LTBP2/TGF-β/Smad3 signaling pathway. This inhibition suppresses TGF-β activation and Smad3 phosphorylation, resulting in reduced cardiac fibroblast proliferation, collagen deposition, and EndMT. The anti-fibrotic effect of Finerenone was further supported by its similar action to the TGF-β inhibitor LY2109761. These findings highlight the therapeutic potential of Finerenone as a cardioprotective agent during DOX-based chemotherapy. They also identify LTBP2 as a promising new target for anti-fibrotic therapy. Further studies are needed to evaluate the long-term safety and efficacy of this approach in patients.

## Figures and Tables

**Table 1:** Primer information for qPCR analysis of expression of target genes.

Primers	Species	Primer Sequences
Col I-Forward	Mouse	TGCTGGTGACCATGATGCTT
Col I-Reverse	Mouse	TCGAGGTTCCCCACAATTGG
Col lll-Forward	Mouse	AACTGAAGGTGGACTCTGCG
Col lll-Reverse	Mouse	CGTACCTGGGAGCGAAGATC
LTBP2-Forward	Mouse	CTGCCCCAGTGGTCAAGGTTA
LTBP2-Reverse	Mouse	GCACTATGTTGAGGCATCGGC
Fmod-Forward	Mouse	TGACAATCGCAACCTCAAGTA
Fmod-Reverse	Mouse	TTGTTGTGGTCCAGGTACAG
Thbs1-Forward	Mouse	GGCGCTCCTGTGATAGTCTC
Thbs1-Reverse	Mouse	TGGTTTCCCGTTCATCTGGG
Comp-Forward	Mouse	CGCAGCTGCAAGACGTGAGAGAGCTGT
Comp-Reverse	Mouse	CCGAATTCCGCTGGTCTGGGTTTCGA
β-actin-Forward	Mouse	GTGACGTTGACATCCGTAAAGA
β-actin-Reverse	Mouse	GCCGGACTCATCGTACTCC
snail1-Forward	Rat	AAAGCAAACTGAGGGCTCTGCTCG
Snail1-Reverse	Rat	TTCGGTACCGGAAGCTGTTGCA
snail2-Forward	Rat	ACAATCCACGATGCAGAAGCT
snail2-Reverse	Rat	GGGCCTTGGTCCTTTGAGA
twist1-Forward	Rat	TCTGGACAGCTCCCCATTCT
twist1-Reverse	Rat	CAAGGCTAACCTGGAGAAGATG
twist2-Forward	Rat	GGTCTCCCGAACAATCCACGATGC
twist2-Reverse	Rat	ACCTTGGTTCTTTGAGAGCTGTC
β-actin-Forward	Rat	CTTCCTGGGTATGGAATCCT
β-actin-Reverse	Rat	TCTTTACGGATGTCAACGTC

## Data Availability

Data supporting the findings of this study could be obtained from the corresponding authors upon reasonable request.

## Supplementary Materials

The following supporting information can be downloaded at https://www.mdpi.com/article/10.3390/biom15121703/s1, Figures S1–S6 and Table S1. The image originality report (figcheck_report20251026). Supplementary Dataset S1 (Differentially Expressed Genes, DEGs). Supplementary file for Gene Set Enrichment Analysis (GSEA).

## Abbreviations

The following abbreviations are used in this manuscript:

Abbreviation	Full name
DOX	Doxorubicin
DIC	Doxorubicin-induced cardiotoxicity
LTBP2	Latent transforming growth factor beta binding protein 2
Fmod	Fibromodulin
COMP	Cartilage oligomeric matrix protein
Thbs1	Thrombospondin 1
TGF-β	Transforming growth factor-β
Smad3	SMAD family member 3
LVEF	Left ventricular ejection fraction
cTnT	cardiac troponin T
CK-MB	creatine kinase isoenzymes
LDH	lactate dehydrogenase
NT-proBNP	N-terminal pro-B-type natriuretic peptide
TNF-α	tumor necrosis factor-alpha
EndMT	endothelial-to-mesenchymal transition
Col I	collagen I
Col III	collagen III
PSR	picro sirius red stain
NRCFs	neonatal rat cardiac fibroblasts
HUVECs	human umbilical vein endothelial cells
KEGG	kyoto encyclopedia of genes and genomes
GSEA	Gene Set Enrichment Analysis
