# Supplementary material for: Targeting LTBP2 Reveals a Novel Anti-Cardiac Remodeling Mechanism of Finerenone Against Doxorubicin-Induced Cardiotoxicity
Source: Biomolecules. 2025 Dec 5;15(12):1703. doi: 10.3390/biom15121703 (PMC12730392; doi:10.3390/biom15121703)
Supplement: Supplementary file 1 [file biomolecules-15-01703-s001.zip › figcheck_report20251026.pdf]

Figcheck Report

Download Date: 2025-10-26
ID: 2022283020104@whu.edu.cn

RECORD SUMMARY

- Title: .pdf
- Type: PDF
- Size: 1 MB
- Created Date: 2025-10-26
- Analysis Result: Saved 0 of 129 result(s)

CHECK ITEMS

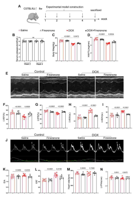
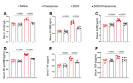
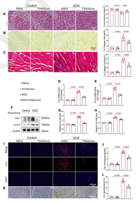
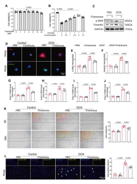
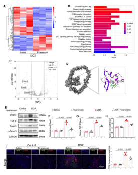
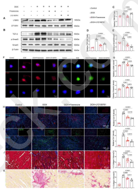

www.figcheck.com

DUPLICATION RESULTS

The above images did not detect duplicate issues this time.

www.figcheck.com

Kind Reminder:

1. Figcheck is only used to improve the efficiency of detecting image duplication, and does not make any recommendations or directions.
2. The Figcheck report is designed to present image pairs selected by the author. The proportion of selected images can be viewed in the "RECORD SUMMARY." The PDF report displays up to 30 entries. The complete results of this test (including false positives, duplicates under normal circumstances, etc.) can be downloaded by clicking **here**. The link will expire in 3 days.
3. This report is generated based on the image recognition of artificial intelligence algorithms. Similar images may be meaningless, such as icons, zooming, fluorescence images, etc. Please judge according to the academic scenario.
4. The final interpretation of this report belongs to the Figcheck team.
5. If you have any questions, please contact the official email admin@figcheck.com.

www.figcheck.com
