# Supplementary material for: Targeting LTBP2 Reveals a Novel Anti-Cardiac Remodeling Mechanism of Finerenone Against Doxorubicin-Induced Cardiotoxicity
Source: Biomolecules. 2025 Dec 5;15(12):1703. doi: 10.3390/biom15121703 (PMC12730392; doi:10.3390/biom15121703)
Supplement: Supplementary file 1 [file biomolecules-15-01703-s001.zip › Figures S1–S6 Table S1.pdf]

Supplemental Materials

All uncropped western blots as well as molecular weight ladders for each blot:

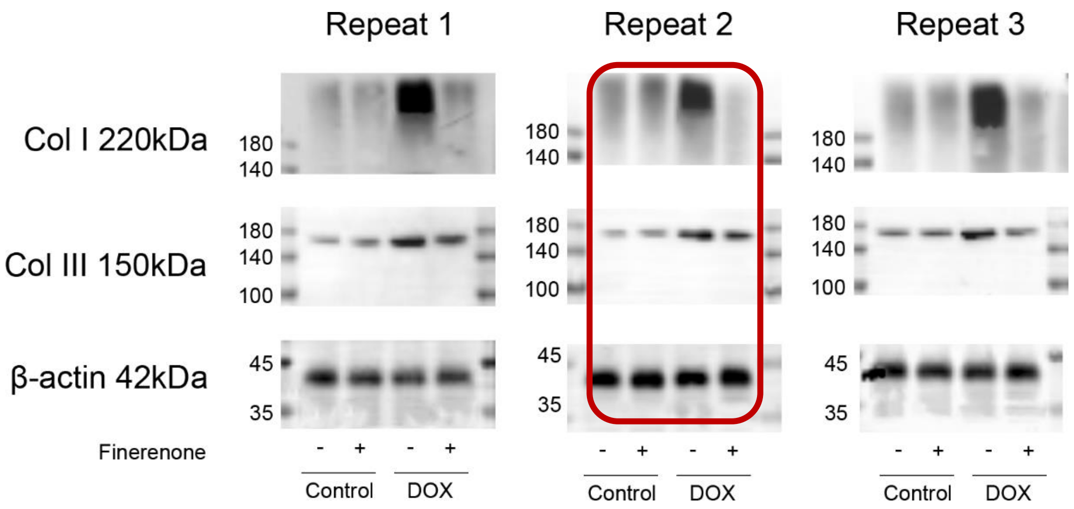

Figure S1. Uncropped Western blot analysis showing protein expression levels of Collagen I (Col I, 220 kDa), Collagen III (Col III, 150 kDa), and β-actin (42 kDa) under different treatment conditions. The blots are representative of three independent experiments. Lanes correspond to the following conditions: Control (no treatment), DOX (doxorubicin treatment), and DOX+Finerenone (combination treatment with doxorubicin and Finerenone). Molecular weight markers are indicated on the left side of each blot. The presence or absence of Finerenone is denoted by "-" for no treatment and "+" for treatment. The red box in Repeat 2 highlights the region corresponding to the cropped Western blot bands used in the main figure, ensuring the accuracy and reproducibility of the data presented.

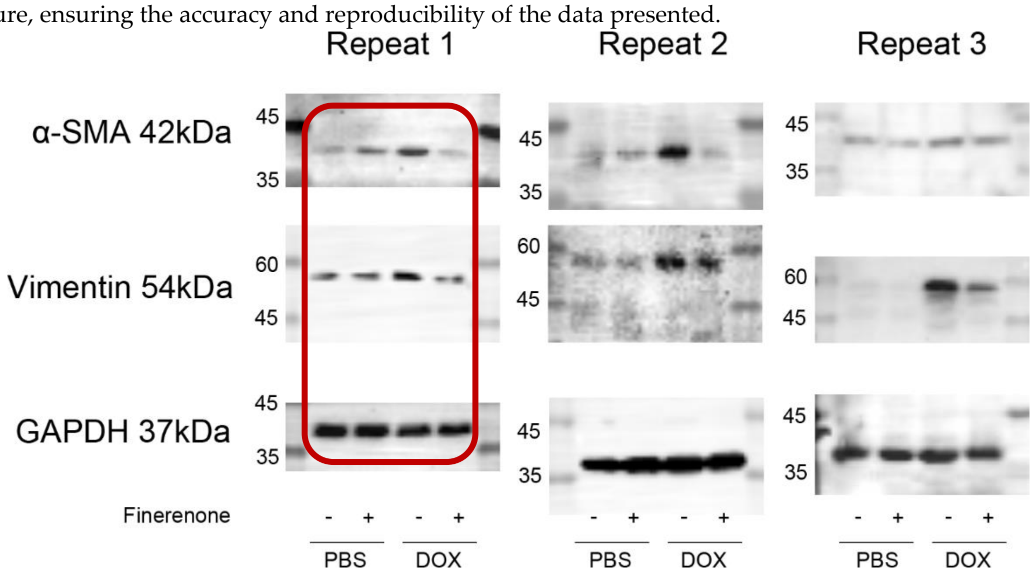

Figure S2. Uncropped Western blot analysis displaying the protein expression levels of α-SMA (42 kDa), Vimentin (54 kDa), and GAPDH (37 kDa) across different treatment conditions. The blots represent three independent experiments. Each lane corresponds to the following conditions: PBS (phosphate-buffered saline, no treatment), DOX (doxorubicin treatment), and DOX+Finerenone (combination treatment with doxorubicin and Finerenone). Molecular weight markers are indicated on the left side of each blot. The presence or absence of Finerenone is denoted by "-" for no treatment and "+" for treatment. The red box in Repeat 1 highlights the region corresponding to the cropped Western blot bands used in the main figure, ensuring the accuracy and reproducibility of the data presented.

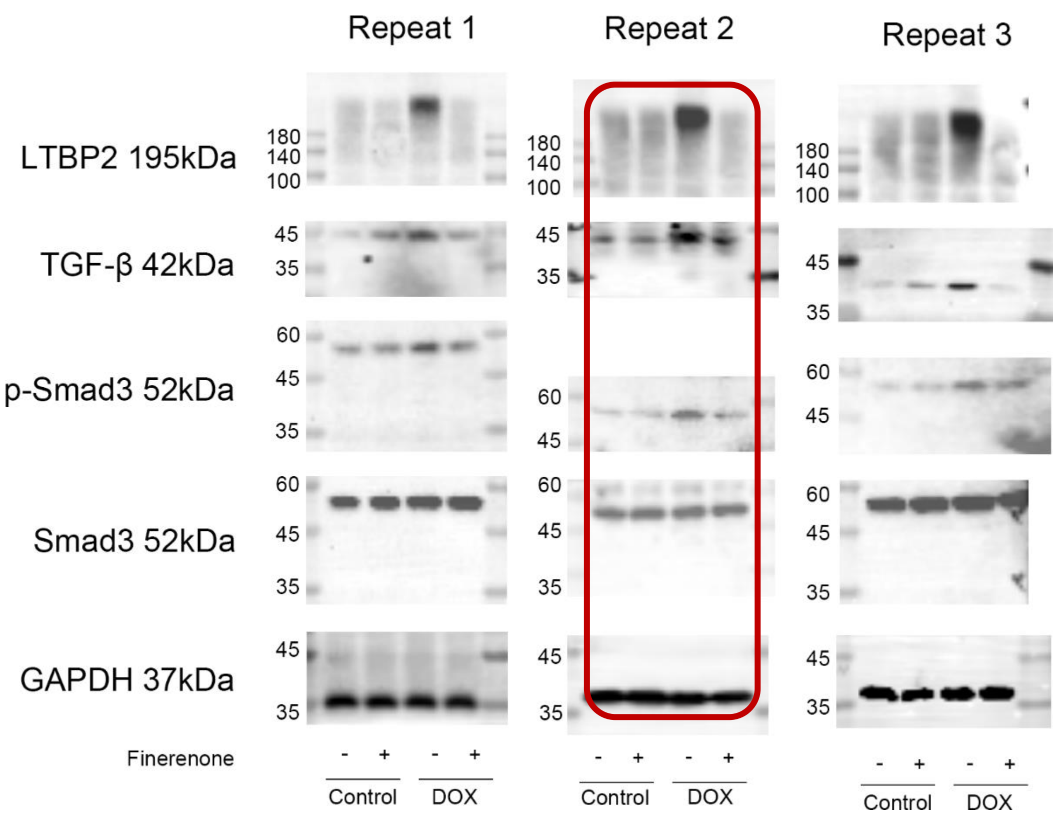

Figure S3. Uncropped Western blot analysis showing the expression levels of proteins LTBP2 (195 kDa), TGF-β (42 kDa), phosphorylated Smad3 (p-Smad3, 52 kDa), total Smad3 (52 kDa), and GAPDH (37 kDa) under different treatment conditions. The blots are representative of three independent experiments. Each lane represents the following conditions: Control (no treatment), DOX (doxorubicin treatment), and DOX + Finerenone (combination treatment with doxorubicin and Finerenone). Molecular weight markers are indicated on the left side of each blot. The presence or absence of Finerenone is denoted by "-" for no treatment and "+" for treatment. The red box in Repeat 2 highlights the region corresponding to the cropped Western blot bands used in the main figure, ensuring the accuracy and reproducibility of the data presented.

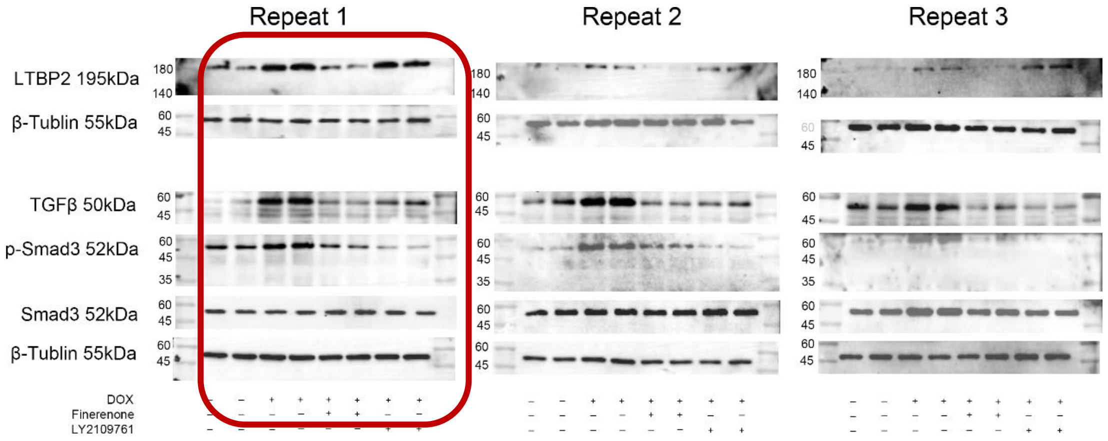

Figure S4. Uncropped Western blot analysis showing the protein expression levels of LTBP2 (195 kDa), β-Tubulin (55 kDa), TGFβ (50 kDa), phosphorylated Smad3 (p-Smad3, 52 kDa), total Smad3 (52 kDa) under various treatment conditions. The Western blots are representative images from three independent experiments. Treatment conditions include DOX (doxorubicin), Finerenone, and LY2109761, as indicated. Molecular weight markers are shown on the left side of the blot. The red box in Repeat 1 highlights the region corresponding to the cropped Western blot bands presented in the main figure, ensuring the accuracy and reproducibility of the data.

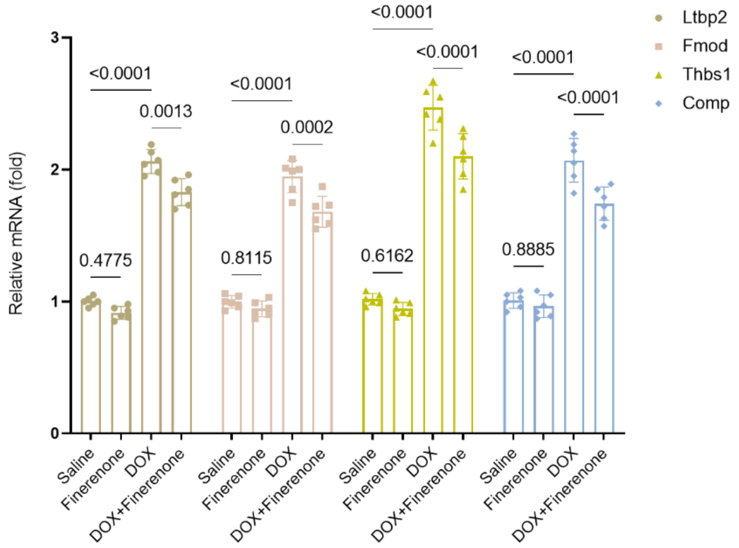

Figure S5. Relative mRNA expression levels of differentially expressed genes in response to treatment with saline, Finerenone, DOX, and the combination of DOX and Finerenone. The genes analyzed include Latent Transforming Growth Factor Beta Binding Protein 2 (LTBP2), Fibromodulin (Fmod), Thrombospondin 1 (Thbs1), and Cartilage Oligomeric Matrix Protein (Comp). All results are shown as the mean ± SEM, p values are indicated, n=6. The fold change values represent the relative mRNA levels normalized to the saline control.

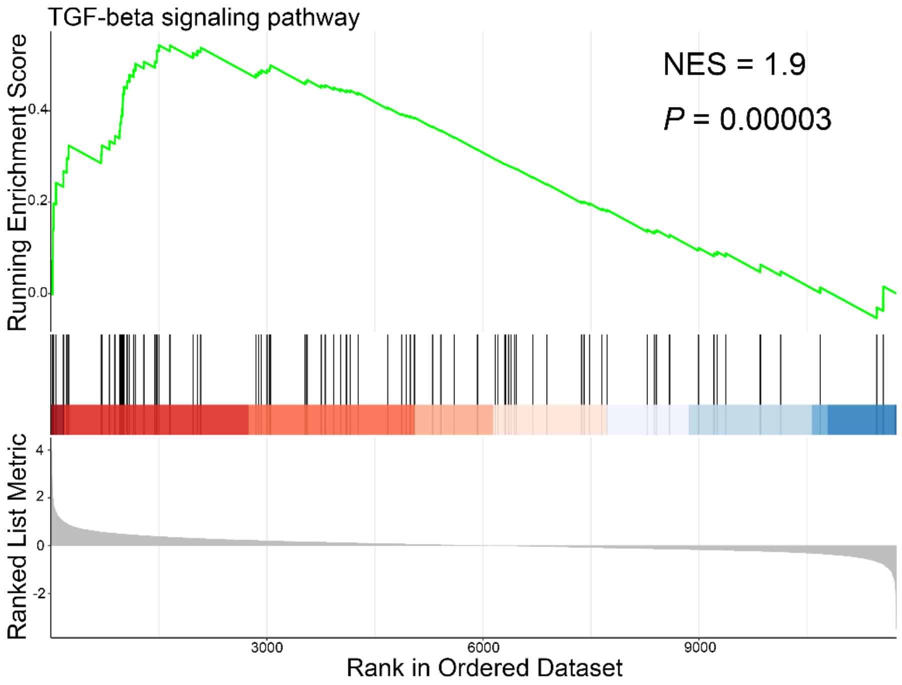

Figure S6. Gene Set Enrichment Analysis (GSEA) of the TGF-beta signaling pathway in the comparison between the DOX treated group and the DOX + Finerenone treatment group. The green line represents

the Running Enrichment Score, indicating the enrichment of the TGF-beta pathway genes in the ranked list. The black bars correspond to individual genes within the dataset, with red and blue indicating upregulation and downregulation, respectively, in the DOX + Finerenone group compared to the DOX + Saline group. The grey curve at the bottom depicts the Ranked List Metric, showing the distribution of gene expression changes across the dataset. NES and p-value are indicated.

**Compound/Protein**	**Docking Binding Energy Score (kcal/mol)**
Finerenone (green in the figure)	-6.5
LTBP2 (purple in the figure)	-7.2

Table S1. Molecular docking results showing the interaction between Finerenone (green) and LTBP2 (purple). The docking binding energy scores indicate the strength of the interaction, with more negative values representing stronger bindings. The table provides the docking binding energy scores for each molecule.
